# Can musical intervention improve memory in Alzheimer’s patients? Evidence from a systematic review

**DOI:** 10.1590/1980-57642018dn12-020005

**Published:** 2018

**Authors:** Shirlene Vianna Moreira, Francis Ricardo dos Reis Justi, Marcos Moreira

**Affiliations:** 1 Graduate Program in Psychology, Department of Psychology, Institute of Human Sciences, Federal University of Juiz de Fora, MG, Brazil.; 2 Unit of Cognitive and Behavioral Disorders (UNICOG), Hospital Maternidade Therezinha de Jesus (HMTJ), Faculdade de Ciências Médicas e da Saúde de Juiz de Fora (SUPREMA), MG, Brazil.

**Keywords:** music, music therapy, memory, Alzheimer’s disease, randomized clinical trials, systematic review, música, musicoterapia, memória, doença de Alzheimer, ensaios clínicos randomizados, revisão sistemática

## Abstract

Treatment with music has shown effectiveness in the treatment of general behavioural and cognitive symptoms of patients with various types of dementia.

**Objective:**

To assess the effectiveness of treatment with music on the memory of patients with Alzheimer’s disease (AD).

**Methods:**

A systematic search was performed on PubMed (Medline), Cochrane Library, PsycINFO and Lilacs databases up to June 2017 and included all randomized controlled trials that assessed memory using musical interventions in patients with AD.

**Results:**

Forty-two studies were identified, and 24 studies were selected. After applying the exclusion criteria, four studies involving 179 patients were included. These studies showed the benefits of using music to treat memory deficit in patients with AD.

**Conclusion:**

To the best of our knowledge, this is the first systematic review focusing on randomized trials found in the literature that analysed the role of musical interventions specifically in the memory of patients with AD. Despite the positive outcome of this review, the available evidence remains inconsistent due to the small number of randomized controlled trials.

## RESUMO

O tratamento com música vem demonstrando eficácia no tratamento de sintomas comportamentais e cognitivos gerais de pacientes com vários tipos de demência.

**Objetivo:**

Avaliar a eficácia do tratamento com música para a memória de pacientes com doença de Alzheimer (DA).

**Métodos:**

Foi realizada uma revisão sistemática nos bancos de dados PubMed (Medline), Cochrane Library, PsycINFO e Lilacs até junho de 2017 que incluiu todos os ensaios clínicos randomizados controlados usando intervenções musicais em pacientes com DA e que avaliaram a memória.

**Resultados:**

Foram encontrados 42 estudos sendo selecionados 24 estudos completos. Após a aplicação dos critérios de exclusão, foram incluídos quatro estudos envolvendo 179 pacientes. Esses estudos mostraram os benefícios do uso da música para tratar o déficit de memória em pacientes com DA.

**Conclusão:**

Até o momento, este é o primeiro estudo de revisão sistemática que utilizou ensaios clínicos randomizados da literatura que analisou o papel das intervenções musicais especificamente na memória de pacientes com DA. Apesar do resultado positivo desta revisão, a evidência disponível permanece frágil devido ao pequeno número de ensaios clínicos randomizados.

Dementia is a public health problem that affects approximately 50 million people worldwide. With the increase in life expectancy, it is estimated that the prevalence of dementia will increase significantly in the coming decades and may triple by 2050.^1^^-^ 3 There are several causes of dementia, and its diagnosis depends on the knowledge of the different clinical manifestations and on a sequence of specific complementary exams. The four most common forms of dementia are Alzheimer’s disease (AD), vascular dementia (VD), frontotemporal dementia (FTD), and dementia with Lewy bodies.^4^ For dementia diagnosis, cognitive or behavioural impairments should affect at least two of the following domains: memory, executive functions, visual-spatial skills, language and personality disorders, or behaviour with symptoms such as mood swings, agitation, apathy, disinterest, and social isolation.^3^^,^ 5

Dementia usually occurs in people over 65 years of age. Age-related physical health problems such as diabetes and hypertension increase the risk of AD and VD. Cognitive reserve can be a protective factor for dementia while minimizing cognitive and functional decline. Factors that increase cognitive reserve such as physical exercise, intellectual stimulation, or lifelong leisure activities are associated with a reduced risk of dementia even in individuals with a genetic predisposition.^2^^,^ 6

National policies highlight the importance of early diagnosis, treatment, and social inclusion to maintaining a good quality of life for people with dementia.^7^ Population studies and international epidemiologic consortia have been investigating the clinical diagnosis of dementia and, dementia of the Alzheimer type has well-established diagnostic criteria for epidemiologic research.^8^ First-line treatment options often involve drugs to slow the progression of the disease and antipsychotic medications to treat behavioural disorders.^1^^,^ 2 Individual strategies in choosing the appropriate dementia medication are highly recommended due to the individual course of the disease and different response to the drugs and their adverse effects.^8^ Music therapy is a non-pharmacological treatment that seeks to minimize symptoms.^9^ Musical interventions are also used by numerous health professionals in patients with AD.^10^^,^ 11

Media interest in this treatment approach has contributed to the public perception that musical abilities represent an “island of preservation” in cognitively impaired people with AD. This intervention can be considered less harmful than pharmacological treatments to improve cognitive functions, mood, and quality of life of these patients.^12^ When music is used acting as therapy, music performs the primary role in the intervention while the therapist is secondary; when music is used in therapy, the therapist takes the primary role and music is secondary.^13^ A music therapist is a qualified professional with the capacity to develop musical interventions adapted to the patient’s experiences and illness. Neuromusical therapy, is a clinical intervention modality of music therapy that acts to promote cognitive rehabilitation of neurological patients.^14^^,^ 15

Studies with musical intervention have demonstrated the efficacy of treatment for the behavioural and psychological symptoms of dementia, such as agitation, irritability, depression, and apathy.^16^^-^ 25 Other studies have investigated the role of music in cognition. In addition, music practice compensates for age-related declines in processing speed, memory, and cognition.^11^^,^ 26 ^,^ 27 However, there is a lack of randomized clinical trials in the area, and in a recent Cochrane meta-analysis^25^ on musical interventions in people with dementia, six articles were found with a total of 257 participants, and there was little effect of treatment on general cognition.

In AD, the ability to recognize music remains relatively preserved,^28^ and patients’ musical memory can be spared, particularly at the onset of the disease.^29^ The human memory system involves a process of coding, storing, and retrieving information.^30^ Musical memory can be defined as the neural coding of musical experiences,^31^ the storage of these experiences, and the subsequent recall of this information. A study by Jacobsen et al. (2015)^31^ involving AD and music indicated a greater preservation of brain areas involved in the processing of music. The authors found that musical memory seems to be partially independent of other memory systems, and in AD, musical memory may be partially preserved. Neural mechanisms and substrates of musical memory involve different anatomical brain networks. Different aspects of musical memory may remain intact while brain anatomy and cognitive functions are impaired.^31^ In addition, regions related to musical memory such as the caudal anterior cingulate cortex and the supplemental motor area showed a minimal level of cortical atrophy and disruption of glucose metabolism compared to the rest of the brain. Therefore, β-amyloid deposition in these regions is at an early stage in the expected course of the development of biomarkers for AD and is relatively well preserved. These results may explain the surprising preservation of musical memory in AD.

Recognizing that musical coding may serve as a mnemonic aid in AD, it is important to review the results of studies that used musical intervention in patients with AD to assess whether these therapeutic modalities were effective in assisting memory. To analyse this question, the authors performed a systematic review focusing on randomized trials.

## METHODS

A search for articles was performed using Boolean operators (“and” and “or”), truncations (music *, Alzheimer * and random *), and the words “music therapy”, “memory”, “cognition”, “Alzheimer disease”, “Randomized Controlled Trial”, and “Controlled Clinical Trial.” We found 42 articles on PubMed (Medline), The Cochrane Library, PsycINFO and Virtual Library (VHL) indexes up to June 2017. We undertook a comprehensive review based on Cochrane protocols.^32^ We excluded 18 articles duplicated in different indexes, giving 24 articles (Table 1).

**Table 1:** Number of randomized trials by year of publication.

Year of publication		Randomized studies
2017		03
2016		03
2015		02
2014		06
2013		02
2012		01
2009		02
2005		01
2004		03
1993		01
Total		24

The selected studies included patients diagnosed with mild or moderate AD who underwent musical intervention with music therapy or with music for memory improvement. The musical interventions were conducted by a trained and qualified professional. The study design was a randomized clinical trial. Studies written in English, Portuguese, or Spanish were selected.

Of the 24 articles found, 20 were excluded. We found four review articles^33^^-^ 36 and an article that used the same population of patients with different methods of analysis.^36^

Of the 15 remaining articles that were excluded, one article represented a study protocol.^37^ Four articles did not evaluate patients with mild or moderate dementia, including the article by Innes et al.^38^ that evaluated patients with mild cognitive impairment (MCI); Sánchez et al.,^39^ who studied patients with severe dementia; and Satoh et al.^40^ (2014) and Tesky et al.,^41^ who evaluated healthy elderly patients. Five papers did not assess memory but used primary measures of mood assessment in music interventions and patients with AD.^42^^-^ 46 Three studies were not controlled and randomized.^47^^-^ 49 The study by Lyu et al.^50^ aimed to evaluate the effect of music therapy on language, memory, and psychological symptoms in patients with mild AD, but was excluded because it was originally written in Chinese. The article by Goudour et al.^51^ used categories of musical instruments *versus* categories of human actions to evaluate semantic memory but did not use musical activities.

**Figure 1:**
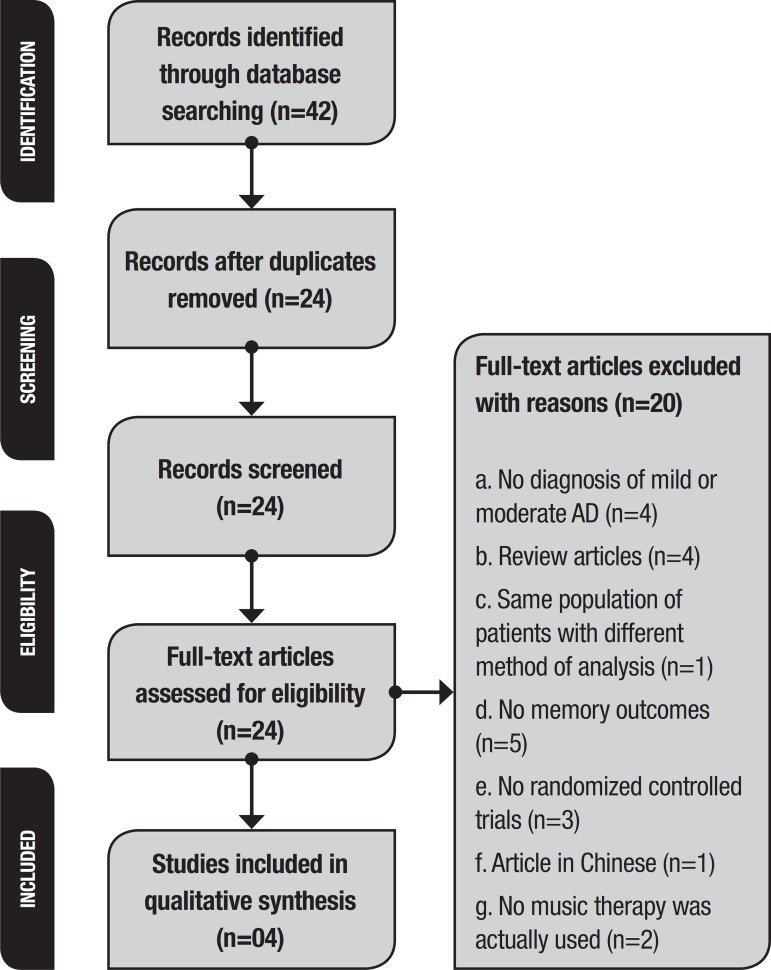
Systematic review flow diagram for the selection procedure.

A flow diagram for the selection procedure is presented in Figure 1. The research design, intervention applied, intervention time, sample size, and evaluations of the studies eligible for analysis are shown in Table 2.

**Table 2:** Characteristics of the included studies.

Author year – country		Type of study		Intervention		Intervention time		Sample		Test battery
Han et al.^52^(2017) – Korea		Multi-center study, double-blind, randomized, controlled-placebo, two-period, crossover		Multimodal Cognitive Enhancement Therapy- MCET *versus* Mock-therapy- MT (simulation therapy)		16 weeks (8 + 8)		N = 32 patients with mild cognitive impairment; N = 32 patients with mild dementia (28 AD, 3 VD, and 1 FTD)		• CDR • MMSE • ADAS-Cog • RMBPC-F • RMBPC-R • GDS • DAD • ADL • IADL
Särkämö et al . ^28^ (2016) – Finland		Controlled, randomized study		Singing *versus* Listening *versus* Usual care		10 weeks		N = 40 patients with AD, 12 = singing, 14 = listening, 14 = usual care; N = 44 other dementias		• MMSE • WMS-III • CERAD • WAIS-III • BNT • WAB • TMT • FAB
Ceccato et al.^53^(2012) – Italy		Randomized controlled trial		Sound Training for Attention and Memory in Dementia (STAM-Dem) *versus* Standard care		12 weeks		N = 51 patients with dementia		• MMSE • Attentional matrices • Digit-span • MPI and MPD • GDS • CMAI • ADL • GMP • SVAM
Lord and Garner^54^ (1993) – USA		Randomized Study		Music *versus* Puzzle *versus* drawing and painting		6 months		N = 60 AD		• Questionnaire

MCI: Mild Cognitive Impairment; CDR: Clinical Dementia Rating; MMSE: Mini-Mental State Examination; ADAS-Cog: Alzheimer’s Disease Assessment Scale – Cognitive Subscale; RMBPC-F: Revised Memory and Behaviour Problems Checklist Frequency; RMBPC-R: Revised Memory and Behaviour Problems Checklist Reaction; GDS: Geriatric Depression Scale; DAD: Disability Assessment for Dementia; ADLActivities of Daily Living; IADL: Instrumental Activities of Daily Living; AD: Alzheimer’s disease; VD: Vascular dementia; FTD: Frontotemporal Dementia; WMS-III: Wechsler Memory Scale III; CERAD: Consortium to a Registry for Alzheimer’s Disease battery; WAIS-III: Wechsler Adult Intelligence Scale III; BNT: Boston Naming Test; WAB: Western Aphasia Battery; (TMT: Trail Making Test; FAB: Frontal Assessment Battery; STAM-Dem: Sound Training for Attention and Memory in Dementia; MPI and MPD: Immediate and Deferred Prose Memory test; CMAI: Cohen Mansfield Agitation Inventory; ADL: Index of Independence in Activities of Daily Living; GMP: Geriatric Music Therapy Profile; SVAM: Music Therapy Activity Scale.

## RESULTS

In a crossover, multicentre, double-blind, randomized, placebo-controlled study, Han et al.^52^ evaluated 64 elderly participants with MCI or mild dementia (patients with AD, VD, or FTD), with a Clinical Dementia Rating (CDR) between 0.5 and 1, and who underwent Multimodal Cognitive Enhancement Therapy (MCET), which included a music therapy programme. The study compared this modality of therapy with a simulation therapy called Mock therapy that consisted of the same intervention time but with daily leisure activities. Primary assessment measures included the Mini-Mental State Examination (MMSE) and the Alzheimer’s Disease Assessment Scale (Cognitive Subscale - ADAS-Cog) to examine the effects of treatment on cognitive function. As a secondary assessment measure, the Revised Memory and Behavioural Problems Checklist (RMBPC) was used to evaluate psychological and behavioural symptoms. The RMBPC provides two types of indexes, i.e., frequencies of problem behaviours in patients with dementia (Revised Memory and Behaviour Problems Checklist (RMBPC-F)) and caregiver reactions to these behaviours (Revised Memory and Behaviour Problems Checklist Reaction (RMBPC-R)). Each index consists of three subcategories, including memory related to problems, depression, and disruption. The Geriatric Depression Scale (GDS) was used to measure the severity of depressed mood, and the Disability Dementia Rating (DDR) was used to measure functional abilities including Daily Living and Instrumental Activities of Daily Living (ADL/IADL). Finally, Quality of Life in Alzheimer’s Disease (QoL-AD) was evaluated. MCET treatment was effective in improving the measures of global cognition (MMSE and ADAS-Cog) and promoted improvements in the RMBPC-F scores. However, no significant differences in the RMBPC-R were identified. The authors concluded that the MCET programme improved global cognition, psychological and behavioural symptoms, and quality of life in people with MCI or mild dementia, suggesting improvements of the activities employed in the care of patients with dementia in South Korea. Notably, the intervention did not affect any specific measure of memory present in the RMBPC-F or RMBPC-R; therefore, it is unlikely that the improvement in MMSE score was due to an effect of MCET on memory. Finally, another important aspect to consider is that musical stimulation was not the main component of the MCET, which was limited to only one hour of musical stimulation per week.

In Särkämö et al.,^28^ the authors trained caregivers to perform daily musical interventions in patients with dementia. Eighty-nine pairs for a total of 178 people, including 89 patients with dementia and 89 caregivers (59 family members and 30 nurses) divided into six blocks, were randomized into three groups of 10 weeks of training and followed up for nine months. The groups participated in singing activities involving familiar songs and occasional vocal exercises and rhythmic movements (n = 30), listening to familiar songs and discussions (n ​​= 29), usual treatment (n = 30), and regular musical exercises at home. The hypothesis was that regular singing would be effective for reinforcing cognitive domains such as attention and working memory and would be beneficial to mood and quality of life. Eighty-four dyads (patient and caregiver) completed the study. All participants underwent a 1.5-hour neuropsychological evaluation that included eight cognitive domains: general cognition (MMSE); orientation (MMSE/Orientation); working memory (MMSE/memory, Wechsler Memory Scale (WMS)-III/Digit span and logical memory); executive function (MMSE/calculation, FAS); long-term memory (WMS-III/logical memory and Consortium to a Registry for Alzheimer’s Disease Battery (CERAD)/word list); executive function (MMSE/calculus and FAS); verbal skills (MMSE/verbal items, Wechsler Adult Intelligence Scale-III (WAIS-III)/Similarities, CERAD/Verbal Fluency, Boston Naming Test, Aphasia Battery (Western Aphasia Battery WAB/sequential commands)); and visuospatial skills (MMSE/copy, WAIS-III/drawing, trails (Trail Making Test TMT/part A). Behaviours such as depression (Cornell-Brown Scale), quality of life (QoL-AD), and caregiver evaluation (Zarit Burden Interview-ZBI and the caregiver’s General Health Questionnaire (GHQ)) were assessed.

The evaluations in the study by Särkämö et al.^28^ occurred before and after the 10-week intervention period and again six months after the intervention. Compared to usual care, singing and listening to music daily improved overall cognition, orientation, attention, executive function, and mood. Concerning memory, the group that engaged in musical activities with singing had better working memory immediately after the intervention than the other groups, but this effect did not persist in the long term (six months later). Because there was a correlation between the frequency of musical activities run by the caregivers in the home and the working memory score six months after the training, it is possible that continuous musical stimulation is necessary to maintain the gains in working memory. With regard to long-term memory, it is important to note that six months after the training, the groups that engaged in some musical activity had a better autobiographical memory for people known in their childhood than the group that was not involved in musical activities.

In 2012, Ceccato et al.^53^ used a music therapy protocol designed for cognitive rehabilitation: the Sound Training for Attention and Memory (STAM-Dem). This multicenter, randomized, controlled, univariate study evaluated the effectiveness of music training in cognitive abilities (attention, memory, and executive function) and behavioural symptoms such as depressive and aggressive states in 51 patients diagnosed with dementia who underwent treatment in different centres in Italy. Their cognitive functions were evaluated using a psychometric battery that included the MMSE, the Attention Matrix, the WMS-III/Digits span, the Immediate Prose Memory Test (MPI), and the Deferred Prose Memory Test (MPD). To assess mood, the GDS was applied, and aggressiveness was measured using the Cohen Mansfield Agitation Inventory. The nurses evaluated the instrumental activities of daily living using the Index of Independence in Activities of Daily Living. The music therapists used two evaluation protocols, namely, the Geriatric Music Therapy Profile (GMP), which evaluated patient competences in the music field, and the Music Therapy Activity Scale (SVAM), which comprises a set of 50 items that explore various aspects of the musical relationship. In this study, a positive effect of the intervention on visual selective attention and verbal episodic memory in the experimental group was observed, indicating that the STAM-Dem protocol can be useful and effective for reactivating functions with cognitive impairment in dementia patients.

Lord and Garner^54^ randomly selected 60 patients diagnosed with AD from a population of 200 patients in a nursing home. The patients were separated non-systematically into three groups, first dividing the women and then the men. The groups consisted of 14 women and six men who participated in different interventions: one group listened to and played American songs of the 1920s and 1930s; another group participated in jigsaw exercises; and the third group participated in usual recreational activities such as drawing, painting, and watching TV. Initially, a questionnaire was developed by the nursing home and researcher in which the questions were based on a list of American Medical Association questions suggested for patients suspected of AD. The questionnaire was administered orally to each participant by the institution’s Director of Nursing, with general information questions that included date, age, sex, and educational level. In addition, questions were asked to evaluate the memory of past personal facts such as, “Where were you born?” and “What was your mother’s name?”. A second portion of the questionnaire assessed patient disposition and social cohesion. A focused observation of 30 seconds was made of the participants for three periods during each activity in the first two weeks of the study, resulting in 36 observations for each patient. During this time, an evaluator (one of the authors) observed the patient’s mood along with their interactive and collaborative propensity on a four-point scale from bad to excellent (zero to four points, respectively). The analysis of the pre-intervention questionnaire showed that there was no significant difference among the groups. Patients received six 30-minute intervention sessions per week for six months. The group that listened to music daily had a greater memory for facts related to their personal history compared to the other groups.

## DISCUSSION

In total, 258 people participated in the studies and were randomly allocated to the control group or experimental group. A positive aspect of the studies was the use of the randomization procedure, but the studies did not explain the type of relationship that the patients had with the evaluators and conductors of the therapy and intervention groups, or the relationship of the conductors of the intervention with the researcher. This is an important data point for the full understanding of the design of each study.

The mean age of the participants was 79.99 years. Only the study by Han et al.^52^ reported the educational level, with a mean of 8.06 years. In the study of Särkämö et al.,^28^ a Likert scale was used to index education in which a score of one corresponded to primary education and seven corresponded to doctoral level, with an average of three points for the singing and control groups and of 2.8 points for the group listening to music. Thus, it was not possible to estimate the educational level of the sample in years. Reporting the level of education in studies is critical because it has been shown in the study by Livingston et al.^2^ that cognitive resilience in adulthood is likely to be increased through education and other intellectual stimuli. In addition, this author stated that lower rates of late dementia are associated with higher education.

The majority of the study participants were women (70.54%). All studies used music therapy or music as a form of intervention. However, one study used music therapy within a broader intervention programme.^52^ The time of intervention varied between the studies^28^^,^ 48 ^-^ 50 from eight weeks to six months, with one to six sessions per week; the service time ranged from thirty minutes to one and a half hours per session. According to the Cochrane meta-analysis,^29^ the therapeutic effects based on music are evident after five sessions. Because all studies included in the present review had more than five therapy sessions, a possible lack of effect of musical intervention in these studies cannot be attributed to a limited intervention time.

Most of the studies used general cognitive screening assessments or tests for assessing specific cognitive abilities such as memory, attention and executive functions, language, and visuomotor skills. Multifunctional batteries were also used, such as measures for evaluations of functional abilities, behaviour, mood, and specific evaluation protocols in music therapy. A summary of the tests used in the studies is presented in Table 3.

**Table 3:** Tests used by type of evaluation.

Evaluation	Tests
Functional evaluation	Index of Independence in Activities of Daily Living and Instrumental ADL (ADL and IADL);Disability Assessment for Dementia (DAD)
Behavioural assessment	Geriatric Depression Scale (GDS); Cohen-Mansfield Agitation Inventory (CMAI)
Cognitive screening	Mini-Mental State Exam - Alzheimer’s Disease Assessment Scale (MMSE)
Multifunctional batteries	Consortium to a Registry for Alzheimer’s Disease (CERAD); Alzheimer’s Disease Assessment Scale – Cognitive Sub-scale (ADAS-Cog); Clinical Dementia Rating (CDR)
Specific cognitive areas	Memory: Revised Memory and Behaviour Problems Checklist Frequency (RMBPC-F) and Revised Memory and Behaviour Problems Checklist Reaction (RMBPC-R); Wechsler Memory Scale (WMS-III); Digits span test; Immediate and Deferred Prose Memory Test (MPI and MPD), assessing semantic memory; Word list memorization test (Consortium to a Registry for Alzheimer’s Disease battery CERAD); Attention and Executive Functions: MMSE calculation; Frontal Assessment Battery (FAB) Attention matrices: evaluates selective focus, concentration, and flexibility; Direct and inverted digit-span. Language: MMSE verbal items; Similarity test of the Wechsler Intelligence Scale (WAIS-III); Verbal fluency test of the Consortium to the Registry for Alzheimer’s Disease battery (CERAD); Boston Naming Test (BNT); Western Aphasia Battery (WAB); Visual-perception: Copy task of MMSE; Cubes of the Wechsler Intelligence Scale III WAIS-III part A (Trail Making Test TMT).
Music therapy evaluation	Geriatric Music Therapy Profile (GMP) scale. Music Therapy Activity Scale (SVAM). Measure improvement related to musical activities.

The studies presented a heterogeneity of cognitive evaluations and different measures. Only the MMSE assessing general cognition was used in most studies. Different approaches to evaluation may make it difficult to detect the efficacy of the treatment outcome. To evaluate the patients, Sarkamö et al.^28^ used 13 different tests on the same day. This type of evaluation can cause stress or fatigue in patients with dementia and may influence the results. The cognitive evaluation performed in the study by Ceccato et al.^53^ evaluated all patients without differentiating the duration and type of dementia. The psychologists responsible for the evaluation encountered difficulties performing the neuropsychological evaluation due to deterioration caused by the dementia or to low levels of education. This evaluation lasted an hour and 15 minutes, in addition to other evaluations performed by nurses and music therapists. Lord and Garner^54^ cited that patients had progressive AD by inferring that they had mild or moderate forms of AD. The authors did not perform conventional cognitive or behavioural assessments; instead, they used a questionnaire developed by the team involving questions from a list of the American Medical Association

The ageing process may be associated with cognitive disorders, and dementia may vary according to the clinical history and type of pathology. Dementia due to AD has the most frequent and well-established diagnostic criteria.^3^^,^ 8 Therapies aimed at relieving symptoms should preferably begin early in the disease stage, when cognitive function is not severely impaired. A two-year delay in the onset of dementia, using predictions based on US incidence studies, could reduce the prevalence of dementia by 23%.^8^ However, most of the studies reviewed did not describe disease duration, stage of AD (mild, moderate, or severe) or even differentiate between types of dementias, which may have different prognoses. For example, Han et al.^52^ analysed MCI patients, who may be clinically identifiable with the initial neuropathological stages of dementia,^8^ patients with mild dementia, 28 patients with AD, three patients with VD, and one patient with FTD. Han et al.^52^ did not differentiate the analyses by pathology, that is, whether the MCI was amnesic or non-amnesic and whether the analysis differed for the different types of dementia. Conversely, Särkämö et al.^28^ reanalysed his data to study clinical and sociodemographic factors that can influence the effectiveness of musical interventions. The author noted in his study that the effectiveness of musical interventions and the outcome of rehabilitation are different for groups of patients with AD, VD, and FTD. The results indicated that the aetiology of dementia, severity, age, and care situations may mediate the cognitive and emotional efficacy of regular singing and/or listening to music. Thus, the heterogeneity of the measures used in the studies hampers comparison and the lack of data on the participants in some studies^53^^,^ 54 does not favour characterization of the sample. It is critical to the advancement of research in the area that basic data on participants such as dementia type, duration, severity of symptoms, and level of education are reported in all studies.

Treatment with music and/or music therapy was investigated in three studies^28^^,^ 53 ^,^ 54 and music therapy integrated into rehabilitation programmes was addressed in one study.^52^ In the study by Han et al.,^52^ we cannot conclude that there was improvement solely due to musical interventions since the MCET programme includes other forms of therapy such as cognitive training, cognitive stimuli, reality orientation, and physiotherapy and reminiscence therapy; music therapy was used only once per week for 60 minutes. In addition, in the study by Han et al.,^52^ the type of musical activity used was not specified; therefore, it is difficult to attribute any specific effect to use of the musical activities. The study by Ceccato et al.^53^ used a music training programme, STAM-Dem, which consists of a progressive series of song sessions used in a sequence of step-by-step exercises to stimulate attention and memory. The other studies used singing and listening in a freer way,^28^^,^ 54 and Särkämö et al.^28^ used popular songs from 1920 to 1960, whereas Lord and Garner^54^ used songs from the 1920s and 1930s when ‘Big Bands’ played on a daily basis. Music therapy is a systematic intervention process that uses different techniques ranging from passive to active musical activities. In this respect, it is important to consider that there is also substantial heterogeneity in the musical intervention, ranging from musical listening techniques to a more systematic use of musical activities with stimulation objectives. Therefore, the effectiveness of the intervention may vary according to the quality of the musical intervention offered.

At this juncture, it is time to consider the main question that guided this systematic review: do musical interventions have an effect on memory in people with dementia? A first point to consider is that the study by Han et al.^52^ does not elucidate this issue because the musical intervention comprised only a part of a broad investigation protocol, thereby precluding inference on any specific effect of the musical intervention in the study. In addition, in the Han et al. study, no effect of the intervention on memory was observed. In other studies, the use of music was the core part of the intervention and involved the use of musical listening, which ranged from listening and singing known songs^28^^,^ 54 to a systematic use of music with the purpose of cognitive stimulation.^53^ In all these studies, the musical intervention had some effect on memory. The questions that arise are as follows: was this effect consistent? What type of memory is most benefited by the intervention?

With regard to working memory or short-term memory, both Ceccato et al.^53^ and Särkämö et al.^28^ used comparable measures of this construct, such as the digit span tasks in direct and inverse order. In this case, the results of the two studies can be considered conflicting in that Särkämö et al.^28^ reported a positive effect of musical intervention on working memory, whereas Ceccato et al. (2012) reported no statistically significant difference between the experimental group and the control group. However, it is important to note that even in the study by Särkämö et al.,^28^ the effect on working memory was limited and did not persist in a further evaluation six months after the intervention. Another aspect to consider is that, in its reanalysis of data, Särkämö et al.^28^ reported that the effect on working memory was moderated by the degree of dementia and was higher in people with mild dementia. Because Ceccato et al.^53^ did not report the degree of dementia of their participants, it is not possible to ascertain whether the difference in the outcome of the studies can be attributed to this factor.

Another measure of memory that can be regarded as comparable between the studies by Ceccato et al.^53^ and Särkämö et al.^28^ is the measure of long-term verbal memory. Both studies included measures that asked participants to repeat a verbally presented passage of text after a time interval ranging from 10 to 20 minutes (the MPD test was used in the study by Ceccato et al.,^53^ and the Logic Memory II test of the Wechsler Adult Intelligence Scale was used in the study by Särkämö et al.).^28^ Again, the results obtained are conflicting between the two studies: this time, the study of Ceccato et al.^53^ reported a positive effect of musical intervention; however, this effect was not statistically significant in the study by Särkämö et al.^28^ A possible explanation for this disparity in results may be due to the task employed by Särkämö et al.^28^ (20 minutes *versus* 10 minutes in the task of Ceccato et al.)^53^ between the study phase and the test phase. This interval may have contributed to a floor effect on the task. After all, if we examine the long-term memory measure (Delayed Memory) in Särkämö et al.,^28^ it can be observed that the task-weighted average amongst the three study groups was only three points out of a total of 35.

Finally, both the study by Lord and Garner and the study by Särkämö et al.^28^ used measures of memory that can be considered to evaluate the autobiographical memory. Both studies used musical listening and observed an effect of this intervention on autobiographical memory. More specifically, in the study by Särkämö et al.,^28^ both the people in the group who only listened to music and the singing group remembered more about the names of people they had known in childhood than the people in the control group. In Lord and Garner’s^54^ study, the participants in the group who listened to and sang songs exhibited better recall of facts related to their personal history than the participants in the two control groups. Thus, the two studies that used more specific measures of autobiographical memory had compatible results. An important fact to consider is that in the reanalysis of Särkämö et al.,^28^ the effect of musical intervention on autobiographical memory was not moderated by any sociodemographic variable, and this is an indication that the effect of musical intervention on this type of memory may be more robust and generalized than other types of memory.

In short, it remains unclear whether musical intervention has an effect on memory, particularly short-term memory and long-term verbal memory, for which conflicting results have been observed.^28^^,^ 53 The most promising results appear to involve autobiographical memory, in which the two studies that more directly investigated this construct reported positive effects of musical intervention on autobiographical memory.^28^^,^ 54 Another positive aspect is that it seems to have made no difference, as far as the effects on memory are concerned, whether the musical intervention was more systematically applied from an intervention programme^53^ or performed from listening.^28^^,^ 54 This is an important point because listening activities or even singing activities are more accessible and can be stimulated in the home by the relatives and caregivers of people with dementia, resulting in a great possibility of preventive activity.

In conclusion, this systematic review aimed to analyse how musical intervention can affect the memory of patients with AD. This is the first review study found in the literature that has this objective. This review demonstrates that there are few randomized studies as of the date of publication. Most of the articles have been published recently, that is, in the last 3 years, which indicates the concern with this type of treatment in the last decade, possibly due to the increase in patients diagnosed with AD in the world population. The articles were published in different countries, including Korea, Finland, Italy, and the USA, but no research was found in Brazil.

The results found in the articles demonstrated that musical intervention may be effective in the treatment of patients with AD. However, the available evidence is still insufficient due to the small number of randomized scientific studies that evaluated memory in patients undergoing music treatment. Despite the limited evidence, it is important to conduct studies with musical interventions supporting the use of music in the complementary treatment for elderly people with dementia due to AD evaluating the impact on cognitive functions and different types of memory. Providing care and rehabilitation for people with AD has become a major challenge for the public health system and for society.^28^ Future studies are necessary with better characterized samples, particularly in terms of the etiology of dementia, duration and severity of symptoms, and the educational level of participants. In addition, the type of musical intervention used should be better described, and sensitive measures of the different types of memory must be included since this is not a unitary construct.
